# Maximum power extraction from solar PV systems using intelligent based soft computing strategies: A critical review and comprehensive performance analysis

**DOI:** 10.1016/j.heliyon.2023.e22417

**Published:** 2023-11-17

**Authors:** Abhinav Saxena, Rajat Kumar, Mohammad Amir, S.M. Muyeen

**Affiliations:** a Department of Electrical Engineering, JSS Academy of Technical Education, Noida, 201301, India; b Department of Electrical Engineering, Dayalbagh Educational Institute, Dayalbagh, Agra, 282005, India; c Department of Electrical Engineering, Indian Institute of Technology (IIT), Delhi, 110016, India; d Department of Electrical Engineering, Qatar University, 5305, Qatar

**Keywords:** FDDL, Intelligent controller, Lyapunov control scheme, MPPT, RCC

## Abstract

This paper shows a comprehensive review on various maximum power point tracking (MPPT) techniques of the solar photovoltaic (PV) cell. It is well understood that power from a solar PV array is sometimes not sufficient, so it is required to extract the maximum power to meet the load demand. In this regard, different techniques were used for comparative analysis like perturb and observe (P & O), fuzzy logic control (FLC), incremental conductance (IC), ripple correction control (RCC), artificial neural network (ANN), particle swarm optimization (PSO), lyapunov control scheme (LCS), and fisher discrimination dictionary learning (FDDL). The performance of MPPT is also examined under the conditions like effect of shading, irradiance, etc. After reviewing the literature, it has been observed that maximum power at different sets of irradiations is extracted with ANN in comparison to other techniques. Subsequently, the least deviations about maximum power point are attained with IC while comparing with other techniques and FDDL has been found the best technique for attaining the minimum total harmonic distortion (THD). In addition to this, it is also detected that the least switching losses are attained with PSO in comparison to others. To this end, it has been concluded that each method has its significance for the extraction of maximum power from the source and dominance over other methods for smart energy systems. The researchers may find this critical review to be a valuable resource in choosing an appropriate soft computing method for the given parameters.

## Nomenclature

**Symbols**
*e*: Error generated
*ig*: Grid currents
*Rs*: Series resistance
*Isc*: Short circuit current
*Rsh*: Shunt resistance
*Voc*: Open circuit voltage
*Vmpp*: Voltage at the maximum power point
*Vp*: Operating voltage

**Abbreviation**
ABC: Artificial bee colony
AI: Artificial intelligence
ANN: Artificial neural network
CMC: Cascaded converter
CV: DC output
EMI: Electromagnetic interference
FC: Fuel cell
FDDL: Fisher discrimination dictionary learning
FLC: Fuzzy logic control
GA: Genetic algorithm
IC: Incremental conductance
ICT: Information and communication technology
LCS: Lyapunov control scheme
LLF: Linear discriminant functions
MPP: Maximum power point
MPPT: Maximum power point tracking
NB: Negative big
NM: Negative medium
NS: Negative small
OC: Open circuit
OD: Overall distribution
P&O: Perturb and observe
PID: Proportional-integral-derivative
PSCs: Partly shadowing conditions
PSF: Projections of sample features
PSO: Particle swarm optimization
PV: Solar photovoltaic
RCC: Ripple correction control
RES: Renewable Energy Sources
RCC: Ripple correlation control
THD: Total harmonic distortion

## 1 Introduction

Due to modernization and rising population, energy consumption has been rising quickly in the modern world. Numerous non-conventional energy resources have been introduced during the past few decades for simultaneous energy generation along with conventional energy resources to sustainably utilize these resources and to meet the rising energy demand [1]. Among the most common non-conventional energy sources like wind, solar, geothermal, tidal, biogas, solar energy is the most practical source due to its extremely long life, low maintenance requirements, lack of toxic waste, and availability. Maximum power extraction in the context of a solar photovoltaic (PV) system refers to the process of extracting the maximum amount of electrical power from the solar panels under given conditions. However, the amount of power solar photovoltaic (PV) arrays can generate at any given moment depends on various factors, including the intensity of sunlight, temperature, shading, and the efficiency of the system components. Maximum Power Point (MPP) generation should be tracked using an appropriate technique since solar energy production varies with changes in irradiance.

The need to extract the maximum power from the solar photovoltaic (PV) is very important because power extraction varies continuously throughout the day from morning to evening due to varying irradiations. In order to meet the rapidly increasing load requirement, the concept of maximum power extraction from solar PV is introduced. To achieve maximum power extraction, solar PV systems are typically designed and operated in a way that optimizes their performance which involves optimizing panel orientation and tilt, minimizing shading, using high-efficiency components, regular maintenance, battery storage etc. [2].

The most common and impressive form of renewable energy generation is solar PV. Solar PV systems are a renewable energy solution that can reduce electricity bills, lower carbon emissions, and contribute to a more sustainable and environmentally friendly energy mix. The size and capacity of a solar PV system can vary widely, from small residential installations to large-scale commercial or utility-scale systems. The nonlinear power-voltage characteristics of PV array, however, make it difficult to run at the greatest power or obtain peak power at every instant of time. A single pair of power-voltage points on the power-voltage feature has the capacity to deliver the greatest amount of power. As PV generation is an ongoing process, the power-voltage characteristic changes with environmental changes after every brief interval of time. The function of renewable energy sources over energy generation has become a top priority in today's world due to the decrease in conventional energy sources and environmental effects. When contrasted with other sources of energy, solar-based energy production has gained popularity because it is more accessible, environmentally friendly, and its rapidly declining cost. Maximizing power extraction is crucial to ensure that a solar PV system generates as much electricity as possible, making it more cost-effective and environmentally friendly. It allows homeowners, businesses, and utilities to make the most of their solar investments and reduce reliance on fossil fuels for electricity generation [3,4].

Maximizing power extraction using intelligent-based soft computing strategies involves leveraging advanced computational techniques to optimize the performance of the system. This study describes and contrasts various MPPT tracking methods. Implementing these intelligent-based soft computing strategies can significantly improve the reliability, efficiency, and overall performance of solar PV systems, leading to higher energy yields and a more sustainable energy supply. By continuously adapting to changing environmental conditions and energy demands, soft computing-based strategies can enhance the efficiency and performance of solar PV systems. However, the specific approach and algorithms used will depend on the complexity of the system and the available data, resources, and various other parameters. Therefore, this paper provides an appropriate way of selecting the most effective intelligent based soft computing strategy under different working conditions. However, practical challenges related to complexity, data availability, and real-world variability must be carefully addressed to unlock their full potential. Further research and development in this field are needed to refine these strategies and make them more accessible for a wide range of PV installations. The most important challenge part observed with the MPPT techniques are the more deviation around the maximum power, insufficient magnitude of the maximum power, more distortion and ripple content. All these issues will be addressed in the upcoming sections.

The first section of the paper gives a brief introduction of the contents of the paper, Section 2 explains the methodology of five major tracking techniques namely Perturbant Observe, Incremental Conductance, fuzzy logic control (FLC), artificial neural network (ANN), and ripple correlation control (RCC). In Section 3, an analytical comparison between the major techniques along with some minor techniques has been made based on the following performance parameters: The effect of Irradiance, the effect of Shading, Settling time as well as speed of tracking at MPP, Ripple at MPP, Economy, and Applications. Section 4 consists of a comparative analysis of various methods for the performance analysis followed by concluding remarks in Section 5.

## 2 MPPT Modelling and control structure

A detailed description of several MPPT methodologies has been presented in this section.

### 2.1 P & O MPPT

**Fig. 1:**
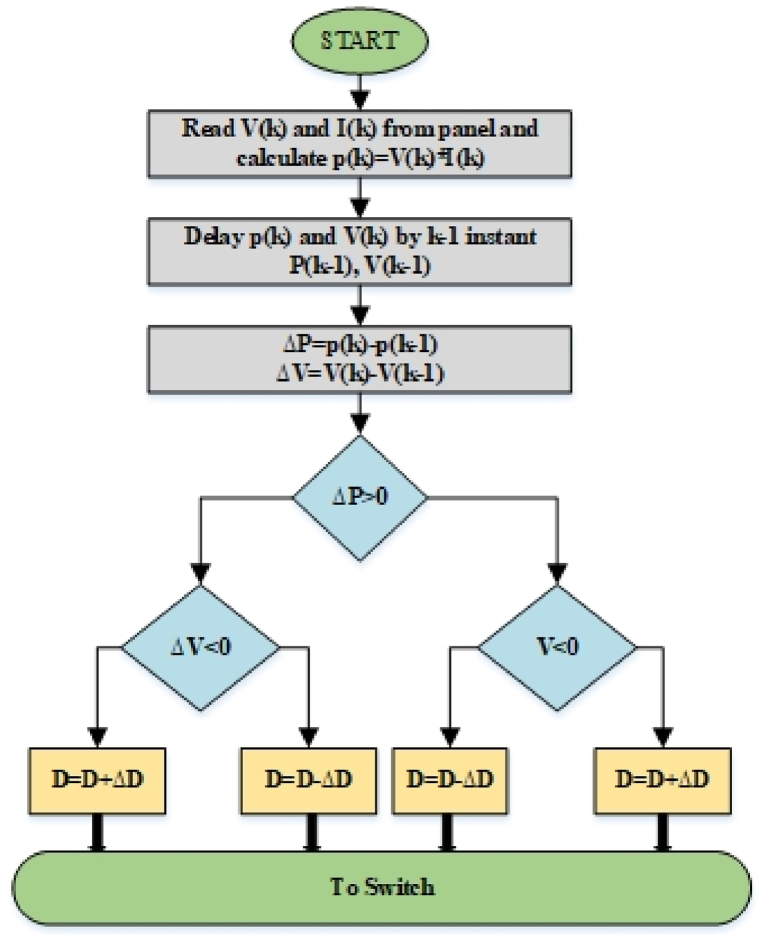
Flowchart of P&O algorithm [1].

It performs a number of cycles in which the solar PV array voltage magnitude is altered by raising or lowering its value, and then it compares the output from the previous cycle to the current cycle. This method is the most generally utilized technique. It is based on the "hill" climbing idea. This allows the control system to adjust the PV array's operating point towards the voltage level where the MPPT is produced [1]. The process of P&O Algorithm can be seen through the flowchart as depicted in Fig. 1 where the duty cycle is being generated for switching the boost converter. A reduced oscillation-based P&O MPPT technique has been proposed in Ref. [2]. It incorporates the dynamic step sizing and a proportional-integral PI controller that can significantly change the duty cycle pulse width modulated signals applied to the DC-DC boost converter while maintaining the structure of the conventional P&O method.

### 2.2 Incremental conductance

Incremental Conductance MPPT overcomes the drawbacks of the P&O MPPT technique. By comparing the incremental conductance to the instantaneous conductance of the solar PV array, incremental conductance calculates the MPP [3],

(1) I/V=ΔI/ΔV

**Fig. 2:**
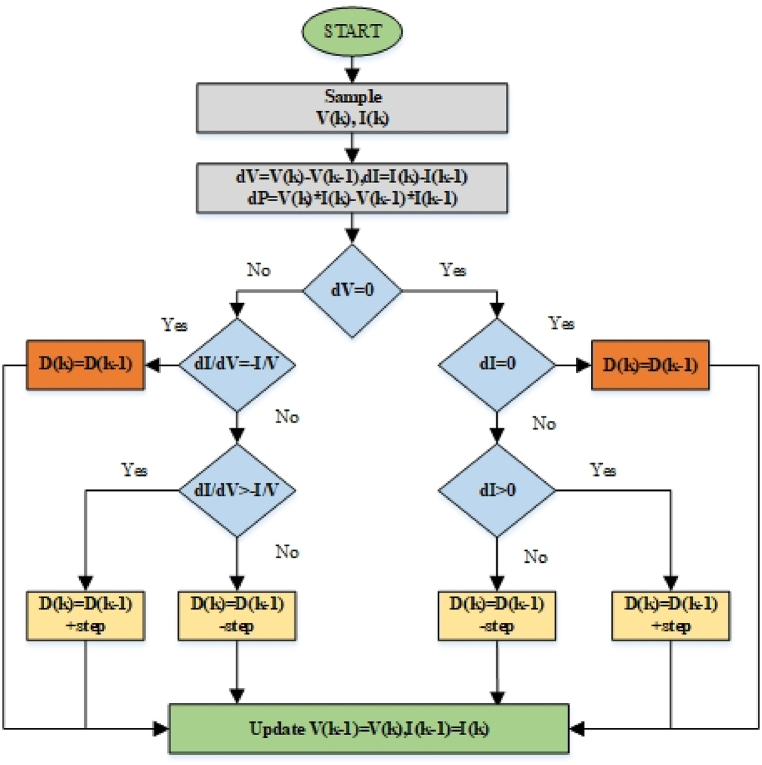
Flow-chart of MPPT comparing by instantaneous conductance [1].

When these two have the same output voltage then it leads to the condition of maximum power point voltage. Until the radiation changes, the controller keeps this voltage constant. Fig. 2 shows the process of incremental conductance in which the duty cycle is being generated for switching the boost converter with less deviation as compared to P&O method. In Ref. [4], the modified IC which is nothing but the error-based IC MPPT, is thoroughly compared to that of the traditional P & O and IC techniques. Regarding the PV system's on-grid use, the highest amount of power collected by modified IC is fed to a 3Φ grid at unity power factor, and the grid current's quality is being watched. Additionally, the electricity acquired by this technique is used to create a PV-diesel generator-based hybrid RES for regions that are either not linked to the grid or have limited fossil fuel resources.

### 2.3 Fuzzy logic control MPPT

Using fuzzy logic, the MPPT method can be controlled which possesses various advantages over other methods. It includes the insight problem with a detailed mathematical representation of the system, and it is more effective at handling nonlinear systems than other approaches [5].

(2) dP/dV=0

(3) dP/dV=d(VI)/dV=IdV/dV+VdI/dV

(4) dP/dV=I+VdI/dV

(5) dI/dV=−I/V

The algorithm of the MPP of the incremental conductance technique is the foundation for the equation's relevance [5].

If dI/dV>0 then Vp<Vmpp (6)

(7) IfdI/dV=0thenVp=Vmpp

If dI/dV<0 then Vp>Vmpp (8)

**Fig. 3:**
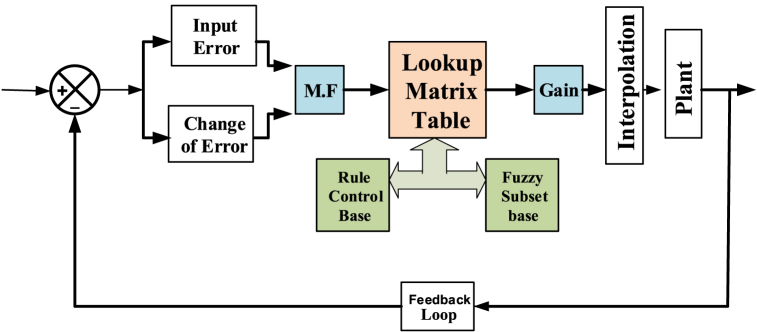
Basic structure of fuzzy logic control [6].

These equations are obtained by comparing the incremental conductance [4] with the operating voltage (Vp) and the voltage at the maximum power point (Vmpp). This process of MPPT is shown in Fig. 3. The fuzzification, rule base, inference, & defuzzification parts comprise the fuzzy logic based MPPT. Creating linguistic variables in accordance with the fuzzy set, which would be found on the fuzzy membership function, is indeed the subject of fuzzy control approach. In fuzzification, the membership function is used to turn the given physical quantity into a fuzzy set. Two physical quantities-based error (e) and change in error (de), which are stated in the direction of MPP are dependent upon the set of if-then situation of the controlled parameter that make up the rule base [5].

(9) $$\Delta I=\frac{\left(P\left(k\right)-P\left(k-1\right)\right)}{\left(V\left(k\right)-V\left(k-1\right)\right)}$$

**Fig. 4:**
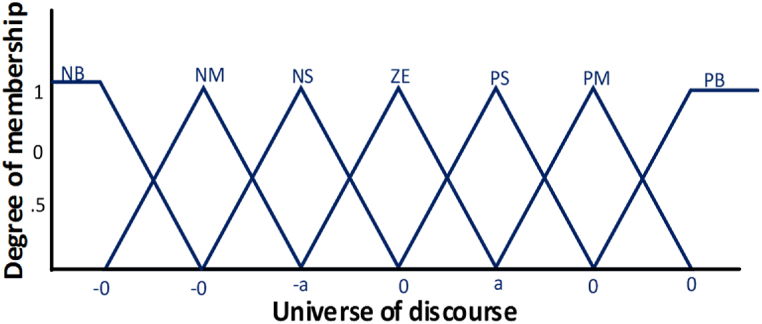
Fuzzy membership [7].

The value of the membership function varies depending on the level of accuracy required to monitor MPP in the solar PV system; typically, its rate ranges from 5to7. In research [5], seven membership values have been taken as: NB (negative big), NM (negative medium), NS (negative small). The nature of membership function is shown in Fig. 4. The fuzzy rules between two inputs as the desired output is shown in Table 1.

**Table 1:** Fuzzy rule between input and output [7].

e/de	NB	NM	NS	ZE	PS	PM	PB
NB	NB	NB	NB	NB	NM	NS	ZE
NM	NB	NB	NB	NM	NS	ZE	PS
NS	NB	NB	NM	NS	ZE	PS	PM
ZE	NB	NM	NS	ZE	PS	PM	PB
PS	NM	NS	ZE	PS	PM	PB	PB
PM	NS	ZE	PS	PM	PB	PB	PB
PB	ZE	PS	PM	PB	PB	PB	PB

In the last step, the linguistic variable is converted back into the numeric values in terms of using the same membership function. The method used for Defuzzification is a center of gravity approach [7]. equation (10) used in the center of gravity method is listed below.

(10) e=I+vΔI/Δv

Where ‘e’ is the error generated. In Ref. [8], the authors proposed the design of a battery charging circuit using an intelligent proportional-integral-derivative (PID) MPPT algorithm using fuzzy logic. Additionally, a solar based reliable PID charge controller is proposed in Ref. [6] for charging EVs batteries with desired ratings.

### 2.4 Artificial neural network MPPT

Artificial neural networks (ANNs) have recently been used in physical systems [[9], [10], [11], [12], [13]]. ANN might be compared to how neurons process information in the human brain. One artificial neuron and aspects relevant make up an ANN. In contrast to other strategies that are based on programs, ANN is an experience and perception methodology that uses data to identify patterns or relationships for training and learning [[11], [12], [13]].

**Fig. 5:**
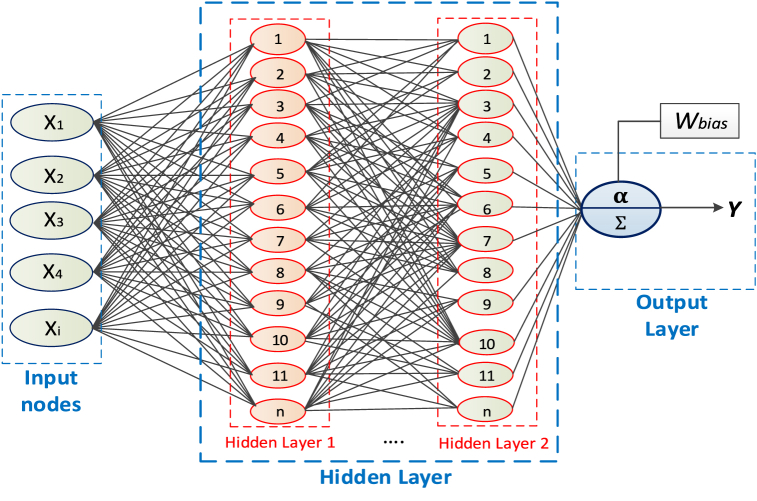
ANN diagram [10].

**Fig. 6:**
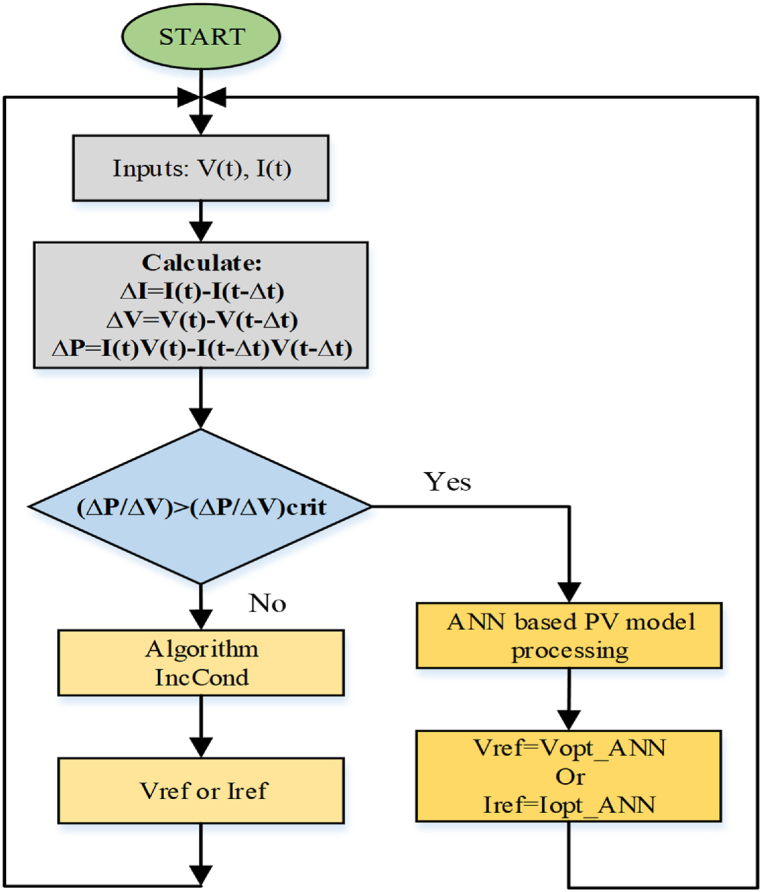
Flowchart of INC based ANN MPPT technique [10].

The design of the ANN system is depicted in Fig. 5. The ANN system has layers such as input-output hidden functions that are based on design and learning rules [14]. PV voltage, power, and environmental information as with any amount of irradiance and temperature are utilized as ANN inputs in solar PV systems to monitor MPPT. Any individual output, such as duty ratio or another, is possible. The system systematically gathers the input data by measuring a variety of factors. The current-voltage parameters for each input are recorded, and MPP is documented for the outputs. Levenberg Marquardt's training tool is used in Simulink to perform the re-training. Since erroneous voltage measurements can occur occasionally, the increment conductance technique is occasionally employed in addition to ANN to precisely track the MPP [14] and this is explained in Fig. 6.

### 2.5 Ripple correlation control

**Fig. 7:**
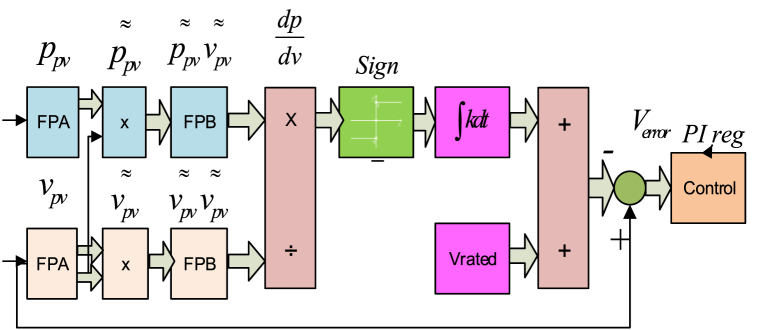
Block diagram of the RCC method [15].

At the IEEE international conference held in July 1996, as one of the programs undertaken in the power semiconductor industry, the latest RCC technique was presented as an MPPT schematic implementation based on the control of the oscillating voltage and the current [[15], [16], [17]]. This oscillation is caused by the flipping of the DC-DC converter proposed inverter, which is called ripple correlation control (RCC). The RCC mechanism is shown in Fig. 7. The change of the energy derivatives over time is linked to the change of the PV committee's current or voltage derivatives, the force behind the slope to zero, and the MPP [[18], [19], [20], [21], [22]].

**Fig. 8:**
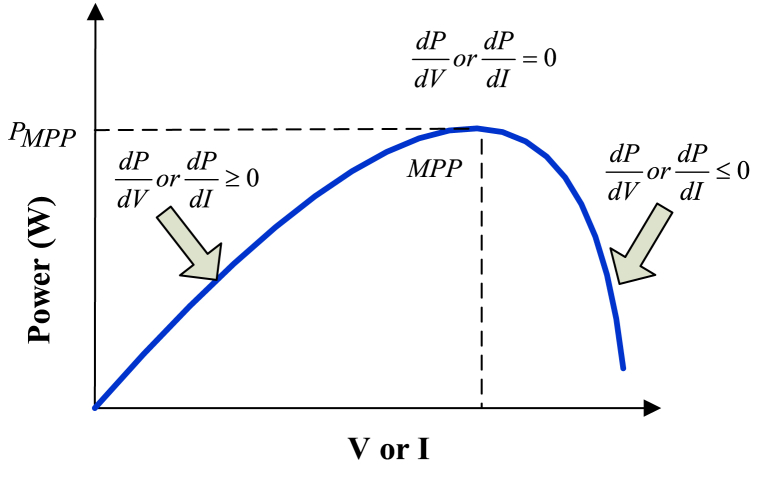
Current or voltage variations in PV panel [18].

A few patterns emerge when examining the curve in Fig. 8. For instance, while increasing voltage as well as current for a predetermined amount of time so that (V>0andI>0) while preserving the pattern P>0, the operating point for a PV panel remains below the maximum power point (V>VforMPPorI>IforMPP). When the tendency is P0, the setpoint is shown to be beyond MPP [[22], [23], [24], [25], [26]]. When the MPP is acquired, it is found that the left MPP is −ve and to the right of this zero, the current or voltage's products are positive. The ripple voltage element is important to lower the array's capacitance in circuitry. The duty cycle can be evaluated using (11) expressions [[27], [28], [29], [30], [31]].

$$\tilde{P_{\mathrm{pv}}}\tilde{V_{\mathrm{pv}}}>0\mathrm{when}V_{\mathrm{pv}}>V_{L}^{*}$$

$$\tilde{P_{\mathrm{pv}}}\tilde{V_{\mathrm{pv}}}<0\mathrm{when}V_{\mathrm{pv}}<V_{L}^{*}$$

(11) $$\tilde{P_{\mathrm{pv}}}\tilde{V_{\mathrm{pv}}}=0\mathrm{when}V_{\mathrm{pv}}=V_{L}^{*}$$

The proposed mathematical equation (11) for the reliability of the RCC method can be inferred for obtaining the switching frequency of the MPPT methods [[32], [33], [34], [35], [36]].

### 2.6 Particle swarm optimization-based intelligent controller

In this control approach, partial shading will decrease power production while increasing the P–V curve's peak numbers. As a result, different line currents will be available from the array's components. To get the most energy from the PV array, the panel should be configured to reduce the row current differential. Further, ‘Su-Do-Ku’ game theory calls for the physical movement of PV array, which may involve lengthy control and complicated linking links. As a result, this study proposed a physical location replacement based on connected PSO modules. This approach keeps the PV arrays tangible placement with the same approach while changing their electrical connections. The outcomes of the PSO technique for electric panel reconfiguring for various shades are contrasted with those of the TCT, Su Do Ku, as well as Genetic Algorithm (GA) approaches currently in use. According to the research, the suggested method can provide a pretty consistent dispersion of shadow over the panel. As a result, the intensity of shadows in a specific location is reduced, increasing power output. Additionally, the disparity in row current is significantly reduced, improving the PV array's I–V characteristics [37].

A detailed comparison of maximum power extraction from PV array with traditional methods and intelligent techniques is illustrated in Ref. [38]. An effort is made in this study to implement improvements to the PSO approach for MPPT, with a focus on initial value selection. The capacity to measure the worldwide maximum output precisely under changing environmental conditions with faster dynamic reaction, negligible steady-state oscillations, and easy implementation are some of this method's significant advantages [39].

The detailed computational modeling for the array's current-voltage distributions for energy yield is given, and they appear to favor cross-tied configurations [40]. Globally, shading issues have been identified in PV applications. This work presents a thorough analysis of the possible linkages among the arrays of a shaded solar field and how these affects generated PV power in order to address this problem. As a consequence, by the use of actual connection laws, a direct relationship between both PV modules connections and their output of electricity is presented [41].

The entire cross-tied connectivity of solar modules is proposed in this study as a novel Zig-Zag configuration to reduce partial shading losses and hence improve power generation. According to simulation results, the new method of reorganization reduces the count of multiple local maxima in power-voltage characteristics, further simplifying the algorithm for tracking the global MPP. Investigation of the system's performance for five distinct shading patterns [42].

PV arrays that are partially shaded have a lower energy output, and their P–V characteristics have several peaks. The losses that result from partial shading vary on the shade pattern, PV array structure, and the actual placement of shaded panel in the module despite of being proportionate to the shaded area. In this study, a method for configuring the array's modules is presented that will increase the array's ability to generate electricity during partial shading [43].

Making PV systems energy efficient is a difficult task. Partly shadowing is one of the primary causes of the decline in PV power. This study offers a fixed connectivity system for PV arrays that increases PV power under various shading scenarios. The proposed method makes it easier to spread the effects of shading across the full array, which lowers the switching loss caused by shading. The findings from MATLAB/SIMULINK are shown to demonstrate that the power fetching from the PV arrays under partial shade conditions is enhanced. The effectiveness of the system is evaluated under various shading cases. To use a lab experimental set-up, experimental results are offered to support the suggested approach. Additionally, a contrast is conducted between the proposed 5x5 PV array plan and the electric array reconfiguring design [44,45].

To lessen the impact of shadowing on PV panels, this research suggests an adaptable reconfiguration strategy. A prototype control technique that boosts the solar PV array's power production connects a solar adaptive bank to a defined area of the system via a switching matrix. Real-time control algorithms are applied. The recommended rearrangements are demonstrated using an experimentally reconfigured PV system with a resistive load [46].

To increase energy output as the solar panel's working circumstances change, this study applies a dynamic electrical array reconfiguring technique to the grid-integrated PV system that relies on a plant-oriented configuration. By adding a programmable switching matrix in among both the PV system and interfacing converter, the EAR approach enables the electrical connection of the accessible PV system. The PV system demonstrates an identity for real-time based adjustment to the PV system operator with internal and external circumstances as a result, which enhances the game's ability to extract energy. Experimental findings are presented to support the suggested strategy [47].

### 2.7 I–V characteristics for maximum power extraction from various modules

**Fig. 9:**
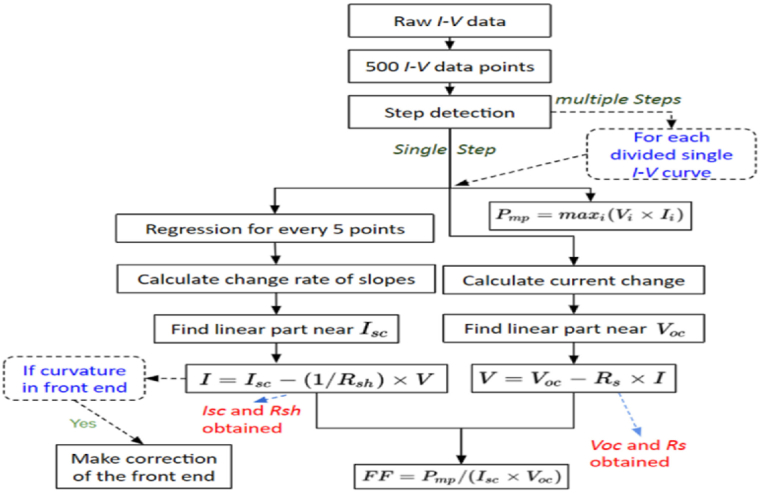
Characteristics of I–V module in PV panel [42].

In order to obtain characteristics from I–V-curves as well as determine the repeatability of all I–V traced points, an information I–V extracting features approach has been devised in this study. To illustrate how this strategy can be used in real-world settings, three separate datasets have been used. Additionally, a simulation analysis has been performed to operate the precision and repeatability of the derived I–V characteristics from a single product. While series resistance (R_s_) estimation displays predicted inaccuracy, the suggested technique works exceptionally well in the estimation of I–V features for short circuit current (I_sc_), shunt resistance (R_sh_), and open circuit voltage (V_oc_). Each value is calculated with a very high degree of reproducibility. Because of this, the data-driven I–V feature extraction method provides a reliable, quick, and data analysis procedure that generates an extraction process for characterizing massive volumes of PV module I–V data [42]. The detailed procedure of extracting the maximum power from I–V modules [48] is illustrated in Fig. 9.

In order to determine the extremely dependable and resilient method(s) of cell description under operational conditions, this paper reviews five distinct approaches for calculating the lump series resistance (RS) of PV cells. Each I–V-curve contrasts with a darkness I–V-curve, comparing of a Suns-VOC with each I–V-curve, evaluation of 2 or even more I–V curves determined at varying lighting levels, and calculation of the region under each IV-curve are the approaches being considered. With a variety of solar cells, the approaches' accuracy is measured. Second, the approaches to resilience in the occurrence of other FF-limitations (like shunt) are too investigated. A significant enhancement of this method was made as a result of the findings and interpretations of a first examination of tiny 2 × 2 cm^2^ solar cells using the integration method, which was supported by a corresponding point [49].

As a direct measure of performance, the I–V characteristic curve is crucial for solar cells and modules. Because of the strong nonlinear link between the model parameters, deriving the diode model parameters in reverse from the I–V curve is quite difficult. This work disproves the assumption that such a nonlinear problem cannot be precisely solved using linear identification methods [[50], [51], [52], [53]]. By identifying the circumstances of bypass diode turn-on, the authors illustrate the viability of assessing cell-level performance heterogeneity using module-level I–V curves. Evaluation of these curves deviates from standard solar performance models based on diodes. The authors demonstrate that this strategy can make extensive use of statistical and machine learning approaches for enormous datasets and integrate these findings with simulations and laboratory-based tests to give valuable information about the quantum superpositions of a PV cell's interface [[54], [55], [56], [57], [58]]. The detailed and conclusive information of various MPPT methods is shown in Table 2.

**Table 2:** Overall and conclusive information of various MPPT methods.

Methods	Description
P & O [1,2]	MPPT analysis is done for variable solar irradiations with inadequate distortion
Incremental conductance [3]	MPPT analysis is done for fixed solar irradiations with less distortion
Fuzzy logic controller [[5], [7], [8], [6]]	With the different set of fuzzy rules, minimum deviation around MPPT has been observed.
Artificial neural network [[9], [10], [11], [12], [13]]	By training the weights of the network for attaining the least deviation around MPPT.
Ripple correlation control [[15], [16], [17], [18], [19], [20], [21], [22]]	Reduces the ripple and distortion around MPPT
reliability of the RCC method [[32], [33], [34], [35], [36]]	Check the reliability status of the maximum power extraction
Particle swarm optimization-based intelligent controller [[37], [38], [39], [40], [41], [42], [43], [44], [45], [46], [47]]	It includes the intelligent controlling methods like PSO and genetic algorithm which controlled the unknown variables for MPPT
I–V characteristics of various modules [[48], [49], [50], [51], [52], [53], [54], [55], [56], [57], [58]]	Each module has its own I–V characteristics and modules with least deviation around MPPT will be considered.
Data driven method [59]	It is the data collection of different sets of irradiations for MPPT
Neuro fuzzy method [60]	It is the solar driven DC link of DFIG which generates as an additional input of MPPT to meet the grid requirement

## 3 Intelligent controlling methods and effect of solar parameters

These methods comprise the various conventional MPPT methods based on the performance criteria listed below.

### 3.1 Effect of irradiance

P&O algorithm is easy to implement and famous too, but even after a number of several improvised models of P & O, the drawback pertinent to an oscillation of MPP around the optimum point during small variations in irradiations still exist. Moreover, during the fast change in irradiation, it fails to achieve MPP quickly leading to large oscillations and hence resulting in a considerable loss in power [25]. The famous modification came with an improvised P&O technique where the max power is obtained at a certain value of irradiation and temperature, afterward P&O technique is applied at the end of each cycle power values are compared with one in the beginning, and if a difference comes zero then MPP is assumed to be obtained.

**Fig. 10:**
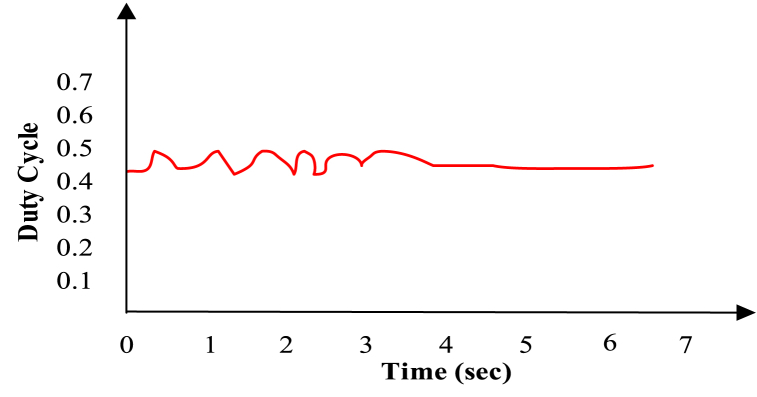
Duty ratio of ANN [23].

**Fig. 11:**
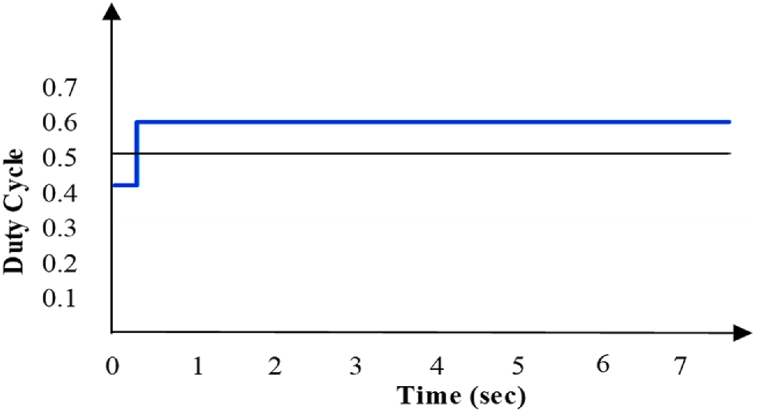
Duty ratio of FLC [23].

The incremental Conductance (IC) technique under different irradiations can maintain a stable operating point in comparison with P&O [26]. ANN technique is much more efficient and modern for effective tracking than P & O under variable irradiation [27]. On further comparison between FLC and ANN, it is observed that ANN regularly tracks MPP with oscillation in duty ratio depicted in Fig. 10 while FLC has a fixed duty ratio depicted in Fig. 11. In the context of solar irradiance control, FLC can be used to adjust the duty cycle of a switching device (e.g., a DC-DC converter or inverter) to maximize the power output of solar panels. FLC relies on fuzzy rules and linguistic variables to make decisions, while ANN leverages machine learning to predict the duty cycle based on historical data and the choice between these two methods depends on factors such as dynamic system complexity, available data, and the specific requirements of the solar power system. Therefore, there is a high voltage fluctuation in ANN than FLC under changing irradiance conditions [27].

### 3.2 Effect of shading

To generate higher voltage for fulfilling the load demand, the series and parallel connections of several PV modules are utilized in some instances, like shading due to clouds, trees, buildings, etc. Also, many PV modules are less illuminated [28]. In case of partial shading in PV system, among many local MPP points, only one global MPP point is formed. The MPPT methods have been formed to trace the global point in all conditions but under partially shaded condition these techniques fail to operate because of many local MPP points are formed while MPPT algorithm tracks the local MPP point rather than global thus an accurate result of MPP is tracked and there is a discrepancy in between the generated power and actual power to be generated. FLC might not adapt quickly to rapid changes in solar irradiance or system parameters, which can lead to a suboptimal tracking performance during dynamic conditions. Therefore, FLC controller is sometimes stuck in local MPP points and gives inaccurate results. Under partially shaded conditions, the P&O algorithm-based MPPT system designed shows multiple peaks in current-voltage characteristics as compared to steady ones under normal conditions which ultimately leads to power loss [29].

To overcome this, some effective modifications were introduced such as a light improvised model which calculates global MPP before P&O cycle for crosschecking, to generate appropriate duty cycles for the converter to obtain optimum results [29].

A novel P&O algorithm is introduced which evaluates the voltage-power characteristics for maximum power and then the P&O runs around the maximum power to extract exact GMPP. Simulation shows efficiency of 84.90 % for MPP tracking [30,31]. Based on a meta-heuristic method and ANN, a thorough and in-depth analysis of MPPT methods in SPV systems under partial shading situations is presented in Ref. [32]. The flowchart and detailed mathematical modeling of thirteen contemporary optimizations and ANN-based global MPP tracking approaches are presented in this review study. In this study, every scheme is evaluated in terms of tracking effectiveness, tracking time, applicability, converter usage, steady-state oscillations, experimental setting, and critical points [32]. The authors in Ref. [33] proposed a thorough analysis of 27 MPPT strategies that are widely used in the PV system. The entire assessment focuses on global MPPT approaches used under partial shade situations as well as MPPT techniques used under uniform solar insolation that varies from time to time. All of the MPPT strategies have been thoroughly compared, along with a brief assessment of their benefits and drawbacks. Additionally, an error-based incremental conductance MPPT with a thorough structure has been developed.

### 3.3 Settling time and speed of tracking (STSPT)

The STSPT speed is a very important aspect of MPPT technique. In a comparative study, it has been found that the speed of tracking for digital techniques like FLC and ANN, is faster than conventional analogue Hill Climbing methods [26]. In P&O method oscillations at MPP are quite high due to fixed step size so the speed of tracking is less with low efficiency [12]. In a comparative study, it has been found that conventional P&O is not only less profound better than its counterpart INC but also considerably slower than it in terms of speed of MPP tracking even under variable irradiation situation [34]. Peak overshoot in ANN is higher than in FLC but oscillating time is less in ANN than FLC so settling time is also less in ANN than FLC at MPP. Although the higher value of overshoot in ANN is high but the stability of ANN is more than FLC with a high speed of tracking [35,36]. In addition to it, FLC doesn't provide a theoretical guarantee of global optimality in the same way that other MPPT algorithms do.

### 3.4 Ripple at MPP

**Fig. 12:**
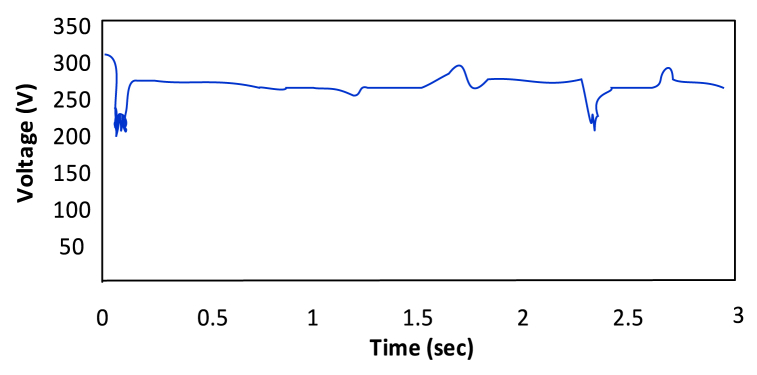
Voltage fluctuation in ANN [30].

**Fig. 13:**
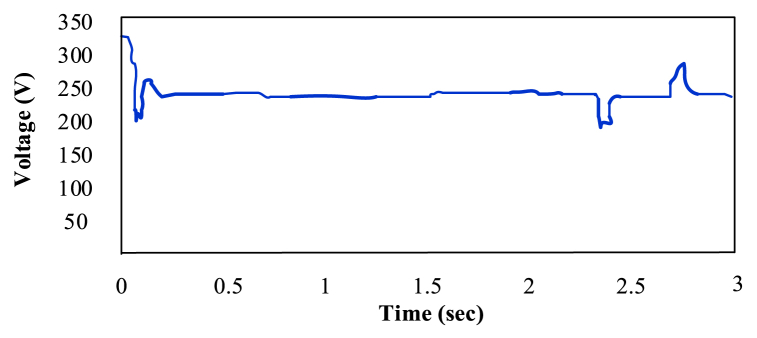
Voltage fluctuation in FLC [30].

Due to the ripples in solar PV voltage, that is twice the frequency of grid, the energy in a power grid that is controlled by load oscillates naturally. This ripple can be used by the RCC approach to track the greatest power point (MPP). RCC is suitable for this technology since ripple naturally occurs in a isloated PV system as a result of the converter switching, which is not the case for other MPP methods like Hill climbing, P&O, and incremental conductance [35]. P&O has a higher ripple content than RCC [36]. Fig. 12, Fig. 13 show that ANN has a larger ripple content than FLC.

### 3.5 Economy and applications

The use of various strategies is discovered to be based on how well they work. For instance, hill climbing techniques like INC and P&O along with several variations are successful in PV, particularly when pretty unusual or when the angle of incidence of the panels is varied. Due to their quick resolution at MPPT, approaches like fuzzy and neural networks are appropriate for solar automobiles, but simple techniques such as an open circuit (OC) and DC output (CV) with fewer restrictions on industrial applications for charging solar streetlamp batteries. The cost factor depends upon the kind (digital or analogue), as electronic procedures deal with more sensors, making them more expensive in comparison because voltage sensors are expensive. As there is no need of sensors for simple methods like OC and CV, hence these are less costly than P&O. The price of INC and makeshift INC approaches is also more expensive. Fuzzy logic & neural networks are two of the costliest digitalization [36].

### 3.6 Fisher discrimination dictionary learning

The nonlinear output properties of solar arrays and tracking of maximum power points methods make fault diagnosis more challenging. The following issues can be resolved using the fault diagnostic model which comprises of an electrical transient time-domain analysis. Existing research on transitioning, however, typically develops its models using sizable, labeled datasets, and some approaches use normalizing techniques with ambient sensors or reference PV panels. The design of the suggested concept involves two stages. Multiple categorizations are initially utilized to identify all types of observed PV array failures. This classifier was trained using tiny, labeled datasets with all fault categories. Then, in the second phase, a lexicon that only contains projections of sample features (PSF) and linear discriminant functions (LLF) is learned to further distinguish LLF and PSF [61].

Similar to other energy production systems, PV systems require maintenance to enhance system performance and to identify the issues before they get out of hand. There are numerous PV monitoring systems available, depending on the output of the plant and its characteristics. Both local on-site monitoring and remote monitoring are possible. It keeps track of production while putting special emphasis on the efficient use of converters and communication tools as well as verification and follow-up. Up to now, a few methods for identifying faults in PV systems and components have been established. The expansion of PV systems, however, has led to ongoing research into more advanced monitoring techniques. This work discusses significant solar system failures [62,63].

The compliance of the L-to-L or L-to-G fault protection standards advised for solar arrays must therefore be thoroughly assessed in light of the different distinctive operational characteristics of PV based generating systems. Additionally, the literature that has already been written mostly examines the standards laid out in the NEC. To assess the efficacy of these standards under various real-world PV operating situations, including 1) changing misfit levels, 2) the influence of MPPT controllers, and 3) variable irradiation levels, extensive modeling, and experimental study have been conducted. In general, this article seeks to evaluate the need for advancements in PV system fault detection criteria [64,65].

This article makes an effort to research, categorize, and evaluate the advanced defect detection techniques that are described in the literature. According to the detection method, each fault detection technique is broken down and evaluated about the following factors: the fault types detected, the detecting time, the sensor requirements, the procedural complexity, the detecting factors, and the protection level attained. The efficiency of complex fault detection methods for protection against the L-to-L, L-to-G, and arc faults, which are the most common in solar based systems, is also evaluated through a compatibility study. Overall, this study is a useful resource for academics looking to increase PV system failure detection capabilities [66].

The use of renewable energy sources is rapidly growing to minimize the effects of global warming and climate change to some extent. Over the last few years, both on-grid as well as off-grid subsystems have installed a significant number of PV systems. Upcoming federal and international regulations, along with the benefits of PV technology, will cause a sharp rise in the number of PV systems. However, in addition to having negative economic effects, the variable nature of solar power generation has other detrimental effects on the functioning of the power grid system, such as instability, reliability, and planning [67].

The use of several innovative metaheuristic methods to optimize the model parameters has reduced the need for additional advancements in this area. Many metaheuristic algorithms have been used for this purpose since then. Even though there are only a few methods available in this area, this article takes the opportunity to examine the existing heuristic algorithm-based parametric extraction methods with a focus on their suitability, accuracy, pace of integration, variety of set variables, and their verifying environment. Aligning for 17 diverse manufacturing solar cells/modules is found using the analysis that was done. A previously unknown gateway between parameter estimation and fault detection in PV systems has been discovered as a result of this review, and it has also assisted scientists in achieving precise, effective, and quick fault detection [68,69].

**Fig. 14:**
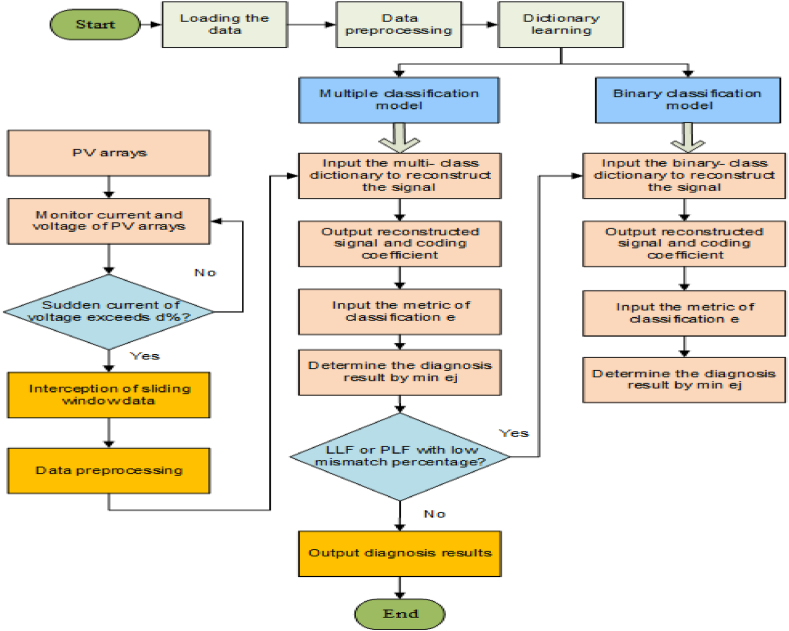
Flowchart explaining the fisherman algorithm [66].

In this study, a PV system damage detection technique is developed along with an analysis of the termination properties of defective PV strings and arrays. The high- and low-voltage damage detection parts are separated by the terminal current-voltage curve of a defective PV array. Then, for each segment, the matching functioning points of good string modules, and faulty modules with a healthy state in an unhealthy string are examined. A defective PV module can be found by probing into several operating locations. For the monitoring of the maximal power point and the adaptive reconfiguration of the array, the fault data is essential. Additionally, by optimizing the positions of the voltage sensors, it is possible to do away with the string current sensors and decrease the overall number of voltage sensors [[70], [71], [72]]. The process of fisherman algorithm is explained through the flowchart in Fig. 14.

The influence of information and communication technology (ICT) on research has been considerably more significant, providing digital tools for the process of data collection, analysis, and result sharing. The dissemination of these new, digital forms of research output has a significant impact on the fundamental structure of the scientific article and demands for adjustments. Embedding 3D visualization tools that are simple to use in web publications is an important illustration of how the digital article format improves scientific communication and helps readers in understanding study findings, the automatic improvement of journal articles using text mining, or another technique is the final subject discussed in this essay [73,74].

The inverter's MPPT technology and the PV array's nonlinear properties prevent traditional protection systems from tripping in certain faults, which lowers system efficiency and raises the possibility of fire dangers. The sequence data of transitory in the time domain during faulty conditions are analyzed and then utilized as the input fault features in this study in order to more accurately detect PV array faults under MPPT circumstances. To depict sequence information visually, the solar array's sequential voltage and current are first converted into a two-dimensional electrical series data graph. Second, a topology for a convolutional neural network (CNN) comprises nine max-pooling layers, nine convolutional layers, and a fully coupled layer is suggested for the identification of faults in photovoltaic arrays. The feature classification and extraction processes are integrated into the suggested model for PV array failure diagnostics. Thirdly, this model leverages raw electrical time series graphs to automatically extract relevant feature representation, obviating the necessity to use artificially created data features for PV failure diagnosis. Additionally, the suggested CNN-based PV fault detection method uses reference panels for normalization and only accepts the PV array's current and voltage array as functional key [75,76].

### 3.7 Lyapunov control scheme

In order to acquire the best power harvest from hybrid renewable energy sources without MPPT, this article proposes a single automated fuel cell (FC) based distribution grid-connected system in which the Lyapunov function-based controller architecture is utilized. With the suggested Lyapunov controller, the inverter is compelled to inject sinusoidal current into the utility grid, execute MPPT, and improve power quality. In contrast to the 2-stage hybrid power system, the greater switching frequency has indeed been decreased in this proposed method by using LCL filter inclusion.

**Fig. 15:**
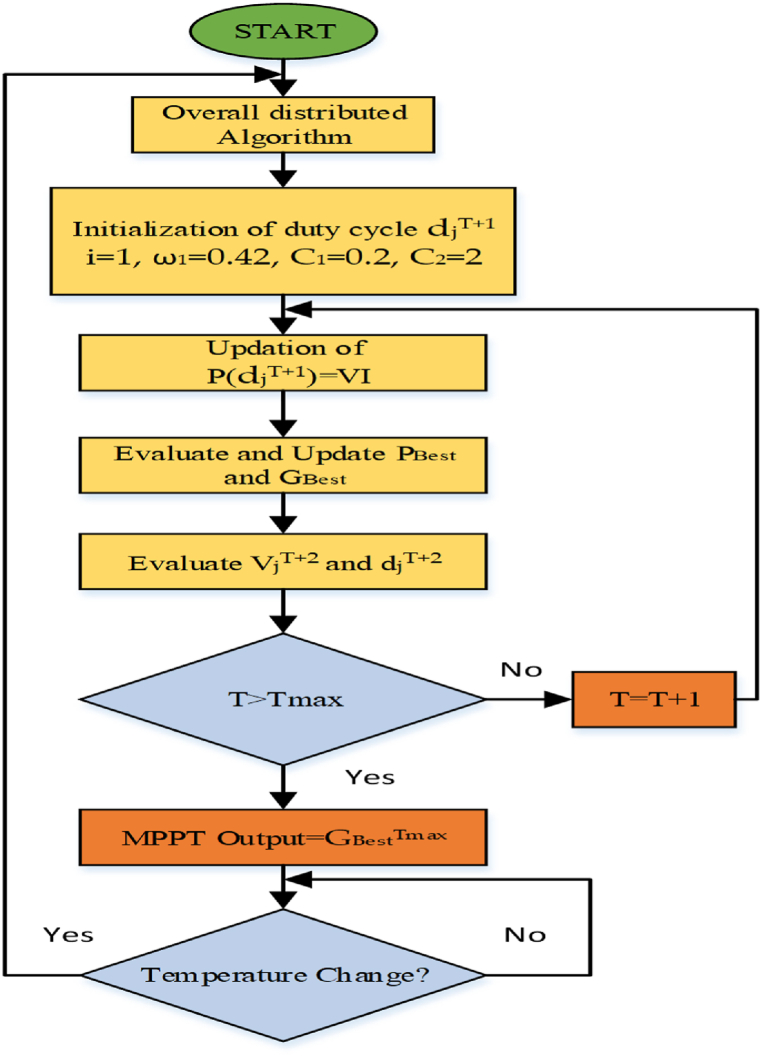
Flowchart explaining the Lyapunov algorithm for maximum power extraction [71].

With its utilized controller, the suggested single service injects steady power into the power system at a cheap cost and with better power quality at the common coupling point. In this research, an integrative CUK converter as well as a mixed overall dispersed particle swarm optimization based MPPT are used [77]. The process of Lyapunov control scheme for maximum power extraction is explained through the flowchart in Fig. 15. The use of the inverter control and grid synchronization with the injection of sinusoidal current is accomplished. The SVPWM controller, which is based on fuzzy logic controllers, adjusts for current error and offers very efficient DC-link use [78].

In this study, a grid-connected PV system that is based on a single-phase and single-stage current source inverter is described. The technology tracks the maximal power point and interfaces the photovoltaic arrays to the grid via a single-stage transformer free conversion. A fuzzy logic controller maintains the maximum power level. The amount of current that is introduced into the grid is managed using a proportional resonant controller. A double-tuned resonant circuit is suggested to reduce the second and 4th harmonics at the converter dc side to enhance power quality and system effectiveness.

It is suggested to magnetize the dc-link inductance after each active switching cycle by cutting off one of the bridge conversion legs using a revised provider modulation technique for the current source inverter [[79], [80], [81]].

To ensure correct results despite the very variable operational conditions generally observed in PV systems owing to solar irradiance, heat, and shadow impacts, among other things, a novel closed-loop adaptable adjustment scheme is developed. The technique can be simply applied to an existing PCC to improve its dead-time rejection capabilities without altering the internal structure because it is simple to build and computationally efficient. A prototype 5-kW PV system's testing findings are presented [80].

This study suggests a cascaded converter (CMC)-based staggered PV connection for PV-grid tie applications. Using a neutral maximum power point tracking controller, it offers a greater depth of operation under partial shading situations with a relatively small filter size and electromagnetic interference (EMI). For the single-phase PV-CMC system, a dq picture control method is being examined for the evaluation of dynamic and strong performance. The performance of PV under partial shade conditions for a standard PV-based centralized and strings inverter in comparison to the proposed PV-CMC technique is also thoroughly compared in order to determine the reliability of the suggested controller [82,83].

The management of solar PV power-generating systems integrated to the distribution network is the topic of this research. Furthermore, to track a solar-PV array's maximum power, a sliding mode control strategy is used. The Lyapunov mechanism control method is created and demonstrated for DC-AC converter architecture to satisfy the needs of active power injections into the distribution grid, modifying grid current (i_g_) approaches towards unity power factor, and adjusting loading cur-rents harmonics. The suggested methods do away with the necessity to modify system parameters while loads and generation are changing. Using its stability analyses, the proposed control strategies' efficacy is determined. Simulation and experimental experiments are used to show how well the proposed control algorithms work with the solar-PV power-generating system under varied operating situations [84].

The suggested system has a number of appealing characteristics, including reduced switching loss, high efficiency, and a small design, which makes it an ideal GCSS for PV systems, in which the voltage output is small and erratic. The architecture of the GCSS elements is provided, along with the equations necessary for the functioning and the derivation of the voltage and current control loop. MATLAB/SIMULINK is utilized to simulate the proposed GCSS. In-depth simulation & experimental data are provided to demonstrate the effectiveness of the suggested GCSS [85,86].

Due to the various localized maximum power points and nonmonotonic PV characteristics of solar photovoltaic systems operating in partly shadowing conditions (PSCs), the efficiency of the current MPPT algorithms for global MPPT is poor, if not inaccurate. In order to swiftly search the area near the global maximum power points, this study recommends a new overall distribution (OD) MPPT approach. To improve MPPT's accuracy, the PSO MPPT algorithm and the OD MPPT algorithm are further combined which is more efficient and accurate than two other artificial intelligence MPPT algorithms via experiments and simulations [87].

For the harvesting of peak power from PV modules, an incorporated CUK converter is suggested. When compared to artificial bee colony (ABC) and particle swarm optimization (PSO) methodologies, the Jaya-based MPPT technique has faster performance and allows for quick PV monitoring with zero deviation all around maximum power point (MPP). Under different operating conditions, the hybrid power system cell with ultra-capacitor as energy storage performs well. By absorbing and delivering power fluctuations, ultra-capacitors offer a quicker dynamic reaction than other energy-storing technologies. Utilizing the dSPACE (DS1104) board, which offers optimum power extraction with steady power support, the hybrid PV -FC based control approaches are experimentally confirmed [88]. The novel shadow distribution (NSD) is an innovative static PV array reconfiguration technique that is proposed in the article [89]. The efficacy of the suggested technique has been assessed and contrasted with the most effective available reconfiguration methods, including zigzag (ZZ), optimal sudoku (OSK), and sudoku (SK), as well as the finest traditional total-cross-tide (TCT) method. Five assessment factors were used to examine the outcome under eight partial shading scenarios.

After doing the exhaustive review of the existing literatures, certain points need to be addressed like variation in the magnitude of maximum power extraction with various methods under the different set of irradiations and different set of shading, a lot of deviation around MPPT in % with various methods under the different set of irradiations and different set of shading, in appropriate THD of solar photovoltaic output with various methods under the different set of irradiations and different set of shading, inappropriate switching losses(W) with various methods under the different set of irradiations and different set of shading have been observed. The overall comparative analysis related to MPPT will be discussed in the next section.

## 4 Performance and comparative analysis of maximum power extraction from various methods

The performance parameters like maximum power, deviation about the maximum power, THD (%), and switching losses are used for choosing the prominent methods for extracting the maximum power. The methods like perturb and observe (P&O) [1], Artificial neural network (ANN) [14], Incremental conductance (IC) [3], fuzzy logic control (FLC) [10], Ripple correction control (RCC) [19], particle swarm optimization (PSO) [87], Lyapunov Control Scheme (LCS) [77], Fisher Discrimination Dictionary Learning (FDDL) [61], Data driven method [59], neuro fuzzy control [60]. are used to extract the MPP under the effect of different set of irradiations and shading. Let's analyze each parameter one by one for all the mentioned methods. Firstly, analyze the maximum power from given methods for a different level of radiation, and the comparative analysis is displayed in Table 3.

**Table 3:** Maximum power extraction comparison with various methods with different irradiations.

Irradiations W/m^2^	P&O [1]	IC [3]	FLC [10]	ANN [14]	RCC [19]	FDDL [61]	LCS [77]	PSO [87]	Data driven method [59]	Neuro fuzzy control [60]
**25**	26	36	49	102	96	87	79	61	99	94
**50**	36	51	75	114	102	91	95	86	94	89
**75**	49	63	81	112	92	79	96	89	95	90
**100**	51	71	92	109	87	91	93	82	96	91
**125**	47	59	79	113	94	86	89	72	97	92

**Fig. 16:**
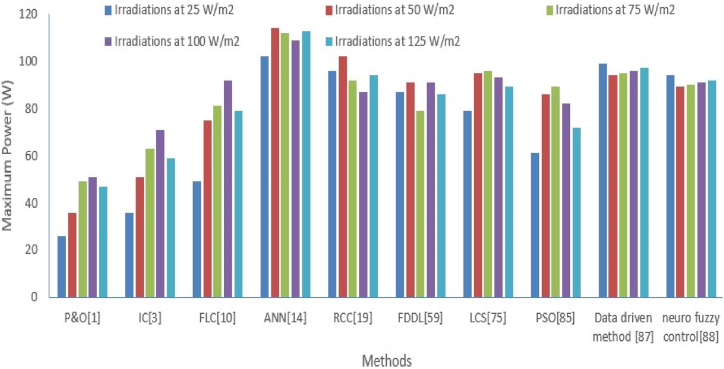
Graphical comparison of maximum power from various methods at different **levels** of irradiations.

In Table 3, Maximum power extraction from solar photovoltaic cell have been analyzed at different level of radiations like 25 W/m^2^, 50 W/m^2^, 75 W/m^2^, 100 W/m^2^, 125 W/m^2^ with methods like P&O [1], Incremental conductance (IC) [3], fuzzy logic control (FLC) [10], ANN [14], Ripple correction control (RCC) [19], particle swarm optimization (PSO) [87], Lyapunov Control Scheme (LCS) [77], and Fisher Discrimination Dictionary Learning (FDDL) [61], Data driven method [59], neuro fuzzy control [60]. A similar type of comparison is illustrated graphically as shown in Fig. 16. It is observed that maximum power extractions are achieved with ANN in comparison to other intelligent methods.

**Fig. 17:**
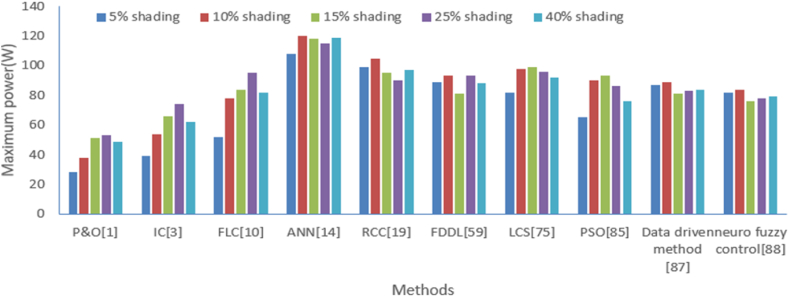
Graphical comparison of maximum power from various methods at different levels of shading.

**Fig. 18:**
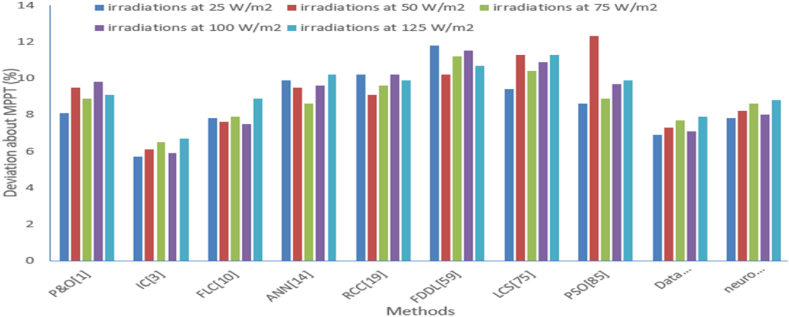
Graphical comparison of deviation about MPPT from various methods at different level of irradiations.

Similarly, maximum power extraction from solar photovoltaic cells has been analyzed with the above-mentioned methods for a different level of shading which is shown in Table 4, and its graphical analysis is shown in Fig. 17. Again, the dominance of ANN plays a significant role in maximum power tracking from other intelligent methods. Now, Deviation about MPPT from solar photovoltaic cell have been analyzed at different level of radiations like 25 W/m^2^,50 W/m^2^, 75 W/m^2^, 100 W/m^2^, 125 W/m^2^ with methods like perturb and observe (P&O) [1], Artificial neural net-work (ANN) [14], Incremental conductance (IC) [3], fuzzy logic control (FLC) [10], Ripple correction control (RCC) [19], particle swarm optimization (PSO) [87], Lyapunov Control Scheme (LCS) [77], Fisher Dis-crimination Dictionary Learning(FDDL) [61], Data driven method [59], neuro fuzzy control [60]. This comparison is illustrated in Table 5. A similar type of comparison is illustrated graphically as shown in Fig. 18. It is observed that the least deviation about MPPT is achieved with IC in comparison to other intelligent methods. Similarly, the minimum deviation about MPPT (%) is achieved with IC in comparison to other methods which is shown in Table 4 and its graphical analysis is shown in Fig. 18.

**Table 4:** Maximum power extraction comparison with various methods under the different level of shading.

Maximum power (W)	P&O [1]	IC [3]	FLC [10]	ANN [14]	RCC [19]	FDDL [61]	LCS [77]	PSO [87]	Data driven method [59]	Neuro fuzzy control [60]
5 % shading	28	39	52	108	99	89	82	65	87	82
10 % shading	38	54	78	120	105	93	98	90	89	84
15 % shading	51	66	84	118	95	81	99	93	81	76
25 % shading	53	74	95	115	90	93	96	86	83	78
40 % shading	49	62	82	119	97	88	92	76	84	79

**Table 5:** Deviation about MPPT comparison with various methods under the different level of irradiations.

Deviation about MPPT (%)	P&O [1]	IC [3]	FLC [10]	ANN [14]	RCC [19]	FDDL [61]	LCS [77]	PSO [87]	Data driven method [59]	Neuro fuzzy control [60]
irradiations at 25 W/m^2^	8.1	5.7	7.8	9.9	10.2	11.8	9.4	8.6	6.9	7.8
irradiations at 50 W/m^2^	9.5	6.1	7.6	9.5	9.1	10.2	11.3	12.3	7.3	8.2
irradiations at 75 W/m^2^	8.9	6.5	7.9	8.6	9.6	11.2	10.4	8.9	7.7	8.6
irradiations at 100 W/m^2^	9.8	5.9	7.5	9.6	10.2	11.5	10.9	9.7	7.1	8
irradiations at 125 W/m^2^	9.1	6.7	8.9	10.2	9.9	10.7	11.3	9.9	7.9	8.8

**Fig. 19:**
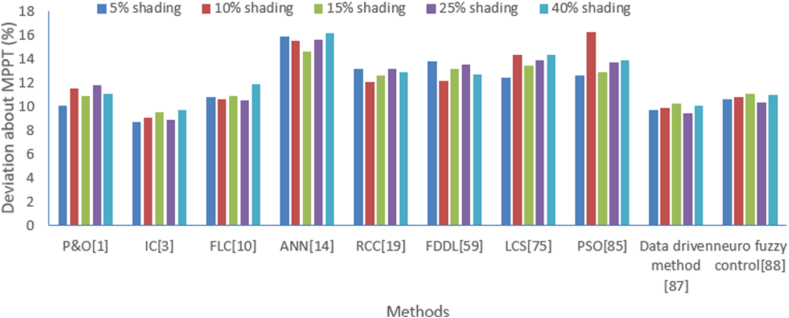
Graphical comparison of deviation about MPPT from various methods at different levels of shading.

In the similar way, deviation about MPPT from solar PV cell has been analyzed with the above-mentioned methods for a different level of shading which is shown in Table 6, and its graphical analysis is shown in Fig. 19. Again, the dominance of IC plays a significant role in achieving the least deviations about MPPT in comparison to other intelligent methods.

**Table 6:** Deviation about MPPT comparison with various methods under the different level of shading.

Deviation about MPPT (%)	P&O [1]	IC [3]	FLC [10]	ANN [14]	RCC [19]	FDDL [61]	LCS [77]	PSO [87]	Data driven method [59]	Neuro fuzzy control [60]
**5 % shading**	10.1	8.7	10.8	15.9	13.2	13.8	12.4	12.6	9.7	10.6
**10 % shading**	11.5	9.1	10.6	15.5	12.1	12.2	14.3	16.3	9.9	10.8
**15 % shading**	10.9	9.5	10.9	14.6	12.6	13.2	13.4	12.9	10.2	11.1
**25 % shading**	11.8	8.9	10.5	15.6	13.2	13.5	13.9	13.7	9.4	10.3
**40 % shading**	11.1	9.7	11.9	16.2	12.9	12.7	14.3	13.9	10.1	11

**Fig. 20:**
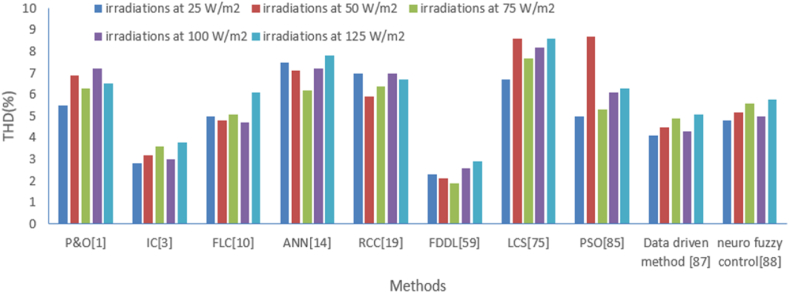
Graphical comparison of deviation about MPPT from various methods at different level of irradiations.

Now, THD of the solar photovoltaic output have been analyzed at different level of radiations like 25 W/m^2^, 50 W/m^2^, 75 W/m^2^, 100 W/m^2^, 125 W/m^2^ with methods like perturb and observe (P&O) [1], fuzzy logic control (FLC) [10], Incremental conductance (IC) [3], Artificial neural network (ANN) [14], Ripple correction control (RCC) [19], particle swarm optimization (PSO) [87], Lyapunov Control Scheme (LCS) [77], Fisher Discrimination Dictionary Learning (FDDL) [61], Data driven method [59], neuro fuzzy control [60]. This comparison is illustrated in Table 7. A similar type of comparison is illustrated graphically as shown in Fig. 20. It is observed that the least THD of solar photovoltaic output is achieved with FDDL in comparison to other intelligent methods.

**Table 7:** THD of solar photovoltaic output comparison with various methods under the different level of irradiations.

THD (%) of photovoltaic output	P&O [1]	IC [3]	FLC [10]	ANN [14]	RCC [19]	FDDL [61]	LCS [77]	PSO [87]	Data driven method [59]	Neuro fuzzy control [60]
**irradiations at 25 W/m2**	5.5	2.8	5	7.5	7	2.3	6.7	5	4.1	4.8
**irradiations at 50 W/m2**	6.9	3.2	4.8	7.1	5.9	2.1	8.6	8.7	4.5	5.2
**irradiations at 75 W/m2**	6.3	3.6	5.1	6.2	6.4	1.9	7.7	5.3	4.9	5.6
**irradiations at 100 W/m2**	7.2	3	4.7	7.2	7	2.6	8.2	6.1	4.3	5
**irradiations at 125 W/m2**	6.5	3.8	6.1	7.8	6.7	2.9	8.6	6.3	5.1	5.8

**Fig. 21:**
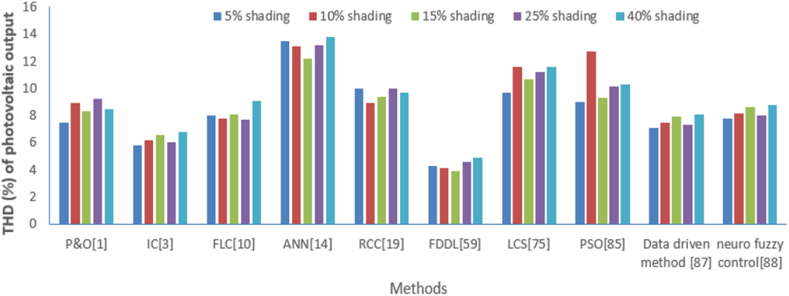
Graphical comparison of THD (%) of solar photovoltaic output from various methods at different level of shading.

In a similar way, THD (%) of solar photovoltaic output has been analyzed with the above-mentioned methods for a different level of shading which is shown in Table 8, and its graphical analysis is shown in Fig. 21. Again, the dominance of FDDL plays a significant role in achieving the least THD (%) of solar photovoltaic output in comparison to other intelligent methods.

**Table 8:** THD of solar photovoltaic output comparison with various methods under the different level of shading.

THD (%) of photovoltaic output	P&O [1]	IC [3]	FLC [10]	ANN [14]	RCC [19]	FDDL [61]	LCS [77]	PSO [87]	Data driven method [59]	Neuro fuzzy control [60]
**5 % shading**	7.5	5.8	8	13.5	10	4.3	9.7	9	7.1	7.8
**10 % shading**	8.9	6.2	7.8	13.1	8.9	4.1	11.6	12.7	7.5	8.2
**15 % shading**	8.3	6.6	8.1	12.2	9.4	3.9	10.7	9.3	7.9	8.6
**25 % shading**	9.2	6	7.7	13.2	10	4.6	11.2	10.1	7.3	8
**40 % shading**	8.5	6.8	9.1	13.8	9.7	4.9	11.6	10.3	8.1	8.8

**Fig. 22:**
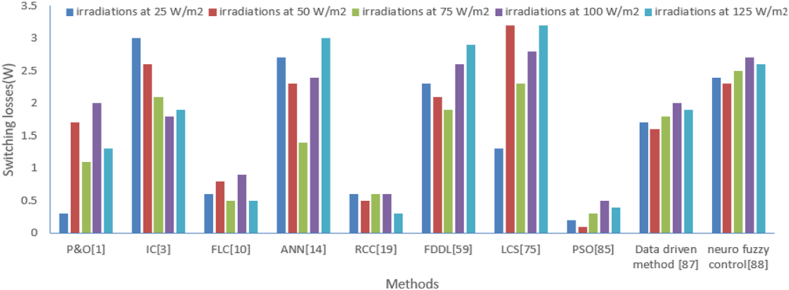
Graphical comparison of switching losses from various methods at different level of irradiations.

**Fig. 23:**
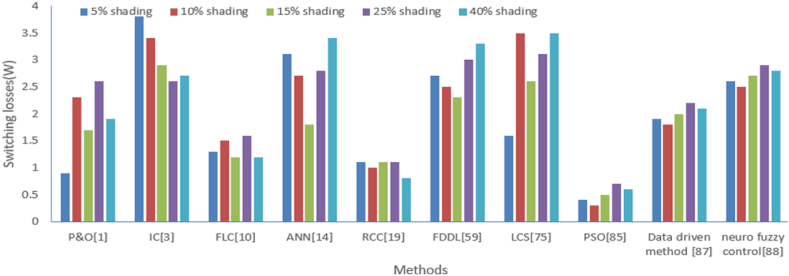
Graphical comparison of switching losses from various methods at different levels of shading.

The switching losses of the solar photovoltaic converters have been analyzed at a different level of radiations like 25 W/m^2^, 50 W/m^2^, 75 W/m^2^, 100 W/m^2^, 125 W/m^2^ with methods like perturb and observe (P&O) [1], Incremental conductance (IC) [3], Artificial neural network (ANN) [14], fuzzy logic control (FLC) [10], Ripple correction control (RCC) [19], particle swarm optimization (PSO) [87], Lyapunov Control Scheme (LCS) [77], Fisher Dis-crimination Dictionary Learning (FDDL) [61], Data driven method [59], neuro fuzzy control [60]. This comparison is illustrated in Table 9. A similar type of comparison is illustrated graphically as shown in Fig. 22. It is observed that the least switching loss is achieved with PSO in comparison to other intelligent methods. Similarly, switching loss has been analyzed with the above-mentioned methods for a different level of shading which is shown in Table 10 and its graphical analysis is shown in Fig. 23. Again, the dominance of PSO plays a significant role in achieving the least switching loss in comparison to other intelligent methods.

**Table 9:** Switching losses comparison with various methods under the different level of irradiations.

Switching losses(W)	P&O [1]	IC [3]	FLC [10]	ANN [14]	RCC [19]	FDDL [61]	LCS [77]	PSO [87]	Data driven method [59]	Neuro fuzzy control [60]
**irradiations at 25 W/m**^**2**^	0.3	3	0.6	2.7	0.6	2.3	1.3	0.2	1.7	2.4
**irradiations at 50 W/m**^**2**^	1.7	2.6	0.8	2.3	0.5	2.1	3.2	0.1	1.6	2.3
**irradiations at 75 W/m**^**2**^	1.1	2.1	0.5	1.4	0.6	1.9	2.3	0.3	1.8	2.5
**irradiations at 100 W/m**^**2**^	2	1.8	0.9	2.4	0.6	2.6	2.8	0.5	2	2.7
**irradiations at 125 W/m**^**2**^	1.3	1.9	0.5	3	0.3	2.9	3.2	0.4	1.9	2.6

**Table 10:** **S**witching losses comparison with various methods under the different level of shading.

Switching losses(W)	P&O [1]	IC [3]	FLC [10]	ANN [14]	RCC [19]	FDDL [61]	LCS [77]	PSO [87]	Data driven method [59]	Neuro fuzzy control [60]
**5 % shading**	0.9	3.8	1.3	3.1	1.1	2.7	1.6	0.4	1.9	2.6
**10 % shading**	2.3	3.4	1.5	2.7	1	2.5	3.5	0.3	1.8	2.5
**15 % shading**	1.7	2.9	1.2	1.8	1.1	2.3	2.6	0.5	2	2.7
**25 % shading**	2.6	2.6	1.6	2.8	1.1	3	3.1	0.7	2.2	2.9
**40 % shading**	1.9	2.7	1.2	3.4	0.8	3.3	3.5	0.6	2.1	2.8

## 5 Conclusions and Scope

This paper presents a thorough analysis of different maximum power extraction (MPPT) methods for solar photovoltaic (PV) cells. It is generally known that not all types of radiation may be effectively absorbed by a solar PV array, hence the maximum amount of electricity must be extracted to fulfill the load demand. Particle swarm optimization (PSO), Lyapunov Control Scheme (LCS), Incremental conductance (IC), fuzzy logic control (FLC), artificial neural network (ANN), ripple correction control (RCC), perturb and observe (P&O), Fisher Discrimination Dictionary Learning (FDDL) were some of the techniques used for comparative analysis at different irradiation level and shading level. The impact of shade, the impact of irradiance, and other factors are also considered in the performance study of MPPT. In comparison to other methodologies, it has been found that ANN extracts the most power from solar PV arrays at various sets of irradiations. Additionally, it is noted that when compared to other techniques, incremental conductance (IC) achieves the least deviations from the maximum power point and Fisher Discrimination Dictionary Learning (FDDL) achieves the lowest THD. Moreover, it has been noted that PSO achieves the lowest switching losses in comparison to other methods. Therefore, it is concluded that each approach has a unique significance for obtaining the greatest amount of power from the source and superiority over other methods for specified electrical parameters.

## CRediT authorship contribution statement

**Abhinav Saxena:** Resources, Writing – original draft, Writing – review & editing. **Rajat Kumar:** Software, Validation, Writing – original draft, Writing – review & editing, Data curation, Formal analysis. **Mohammad Amir:** Conceptualization, Supervision, Validation, Writing – review & editing, Methodology. **S.M. Muyeen:** Investigation, Project administration, Resources, Supervision.

## Declaration of competing interest

The authors declare that they have no known competing financial interests or personal relationships that could have appeared to influence the work reported in this paper.
